# SPR-Optical Fiber-Molecularly Imprinted Polymer Sensor for the Detection of Furfural in Wine †

**DOI:** 10.3390/bios11030072

**Published:** 2021-03-05

**Authors:** Maria Pesavento, Luigi Zeni, Letizia De Maria, Giancarla Alberti, Nunzio Cennamo

**Affiliations:** 1 Department of Chemistry, University of Pavia, Via Taramelli n.12, 27100 Pavia, Italy; giancarla.alberti@unipv.it; 2 Department of Engineering, University of Campania Luigi Vanvitelli, Via Roma n.29, 81031 Aversa, Italy; luigi.zeni@unicampania.it (L.Z.); nunzio.cennamo@unicampania.it (N.C.); 3 Ricerca sul Sistema Energetico-RSE S.p.A.-Via R. Rubattino n.54, 20134 Milano, Italy; letizia.demaria@rse-web.it

**Keywords:** molecularly imprinted polymer (MIP), surface plasmon resonance (SPR), plastic optical fiber (POF), 2-furaldheide (2-FAL), beverages, optical chemical sensors

## Abstract

A surface plasmon resonance (SPR) platform, based on a D-shaped plastic optical fiber (POF), combined with a biomimetic receptor, i.e., a molecularly imprinted polymer (MIP), is proposed to detect furfural (2-furaldheide, 2-FAL) in fermented beverages like wine. MIPs have been demonstrated to be a very convenient biomimetic receptor in the proposed sensing device, being easy and rapid to develop, suitable for on-site determinations at low concentrations, and cheap. Moreover, the MIP film thickness can be changed to modulate the sensing parameters. The possibility of performing single drop measurements is a further favorable aspect for practical applications. For example, the use of an SPR-MIP sensor for the analysis of 2-FAL in a real life matrix such as wine is proposed, obtaining a low detection limit of 0.004 mg L^−1^. The determination of 2-FAL in fermented beverages is becoming a crucial task, mainly for the effects of the furanic compounds on the flavor of food and their toxic and carcinogenic effect on human beings.

## 1. Introduction

The need for low-cost and easy-to-use sensing systems for the rapid screening of food contaminants is constantly increasing. Traditional monitoring techniques are typically based on laboratory analyses of representative field-collected samples; this necessitates considerable time, effort, and expense and the sample composition may change before analysis. Alternatively, portable monitoring systems relying on sensing methods appear well suited to complement standard analytical methods and, also, can be permanently installed at the monitoring sites and can transmit the data remotely. Bio and chemo receptor-based sensors in optical fibers have been shown to be well suited in numerous applications [1,2,3,4,5]. In particular, the optical fiber sensing platforms based on surface plasmon resonance (SPR) [6,7,8,9,10,11,12] allow marker-free detection and have promising merits of low cost, high sensitivity, and small size. In general, the optical fiber for sensing application is either a glass or a plastic one. The prism-based Kretschmann and Otto configurations are the most commonly used in sensing to excite the SPR phenomenon. More recently, systems incorporating plastic optical fibers (POFs) have been introduced [12]. This upgrading makes it possible to reduce the SPR sensors’ cost and dimensions by integrating the sensing platform with small optoelectronic devices (sources and detectors).

For low-cost sensing systems, POFs are especially advantageous due to their excellent flexibility, easy manipulation, great numerical aperture, large diameter, and the fact that plastic can withstand smaller bend radii than glass [12].

The SPR-POF platform developed by our research group is particularly convenient for sensing purposes when it is combined with receptors, as molecularly imprinted polymers (MIP) [13]. It is based on the SPR phenomenon taking place at a multilayer structure realized starting from a planar surface of exposed core POF, embedded in a resin block (D-shaped POF platform). The receptor layer is deposited on the gold-photoresist multilayer [13]. The flat shape of the sensing part is particularly suitable for measurements in a drop, for which no expensive and bulky flow-through cell is required.

The D-shaped POF sensing platform, developed by our research group, has been exploited in several application fields, employing different kinds of receptors, as aptamers, antibodies, metal ligands, and MIPs [13,14,15,16,17,18,19,20]. The MIPs are a class of artificial receptors whose concept dates back to the early 1930s [21,22,23]. Peculiar is the process of MIP preparation, which is based on a template-assisted synthesis [24]. The target molecule is dissolved in a liquid phase together with functional monomers able to coordinate around it both by covalent or non-covalent bonds. Next, the complex is polymerized in the presence of a cross-linking agent. Upon template removal, cavities are left on the polymeric material; they are complementary to the template in shape, size, and position of recognition sites. MIPs often possess recognition properties analogous to natural receptors but have the stability, ease of preparation, micromachining, integrability, and low cost of production, typical of synthetic materials [24,25,26].

MIPs are synthetic solids containing sites functionally and dimensionally complementary to the target molecular structure, similar to the receptor sites in bioreceptors. Moreover, it is important to emphasize that MIPs can be produced as layers in tight contact with the transducing surfaces [13,14]. It must be recognized that in this form, MIPs are different from the usual bioreceptors, for example, antibodies or aptamers, since their thickness can be higher than that of the bioreceptors’ layers, usually constituted by only one or a few molecular layers. This aspect can be very important to reduce the so-called “bulk effect”. In particular, the MIP’s thickness can be modified in function of the real matrices in which the analyte is present; for instance, to exploit SPR-POF platforms to detect specific substances in power transformer oils, very thick MIP layers have been used [14].

MIPs have been found to have many interesting applications for selective separations and as biomimetic receptors in sensing devices, mainly for detection outside the laboratory, because of their robustness characteristics in different conditions (acidity, ionic strength, etc.), low cost, and fast development. In the D-shaped SPR-POF platform proposed by us, an MIP layer can be easily deposited by a drop coating and spinning procedure, as previously described in several cases [13,14,19,27].

In this work, the application of an SPR-POF platform with MIP as a specific receptor for the selective detection of 2-furaldheide (2-FAL) in aqueous solutions for food safety surveillance is presented. The 2-FAL detection in aqueous solutions or beverages, for instance, wine, is becoming a crucial task not only for its relevance in affecting the flavor and aroma [28], but also for its suspected toxic and carcinogenic effects on human beings [29,30,31]. Moreover, furanic compounds have been proposed to assess the aging of food and beverages due, for example, to inappropriate storing conditions [31,32,33,34].

An MIP for 2-FAL has been recently tested for sensing by electrochemical transduction by our group [35], taking advantage of the redox properties of 2-FAL. Here, the same MIP previously investigated is proposed with optical SPR transduction to improve the detection limits while maintaining similar characteristics of low cost and fast development of the biomimetic receptor. The portability of the apparatus is assured by the use of the POFs with a low dimension apparatus. In this study, the effect of the thickness of the MIP layer on the sensor response is investigated, particularly to modulate the SPR resonance wavelength according to the characteristics of the sample, mainly its refractive index (RI).

## 2. Materials and Methods

### 2.1. Chemicals and Instrumentation

Divynilbenzene (DVB, CAS N. 1321-74-0), methacrylic acid (MAA, CAS N. 79-41-4), 2-furaldehyde (2-FAL, CAS N. 98-01-1), and 2,2′-azobisisobutyronitrile (AIBN, CAS N. 78-67-1), were obtained from Sigma-Aldrich. MAA and DVB were purified with molecular sieves (Sigma-Aldrich cod. 208604, St. Louis, MO, USA) before use to remove stabilizers. All other chemicals were of analytical reagent grade. Pure water was obtained by a Milli-Q system (Merck Millipore, Billerica, MA, USA). Stock solutions of 2-FAL were prepared by weighing the liquid and dissolving it in pure water.

The wine-mimicking solution had the following composition: fructose 25 g/L, glucose 25 g/L, tartaric acid 5 g/L, glycerol 1.25 g/L, ethanol 180 mL/L (18% *v*/*v*). (pH = 3.3, *n* = 1.3455 RIU).

The white wine sample (WW) was a white wine in tetra pack purchased in a local supermarket.

**Figure 1:**
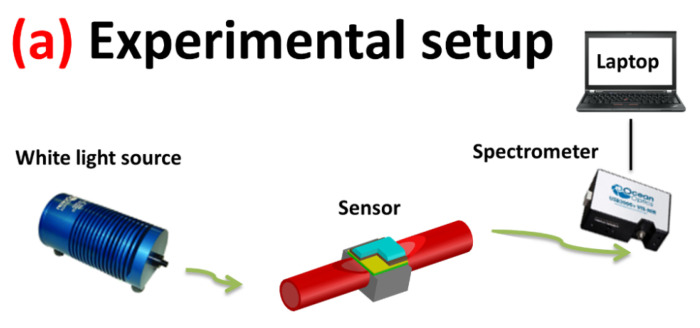

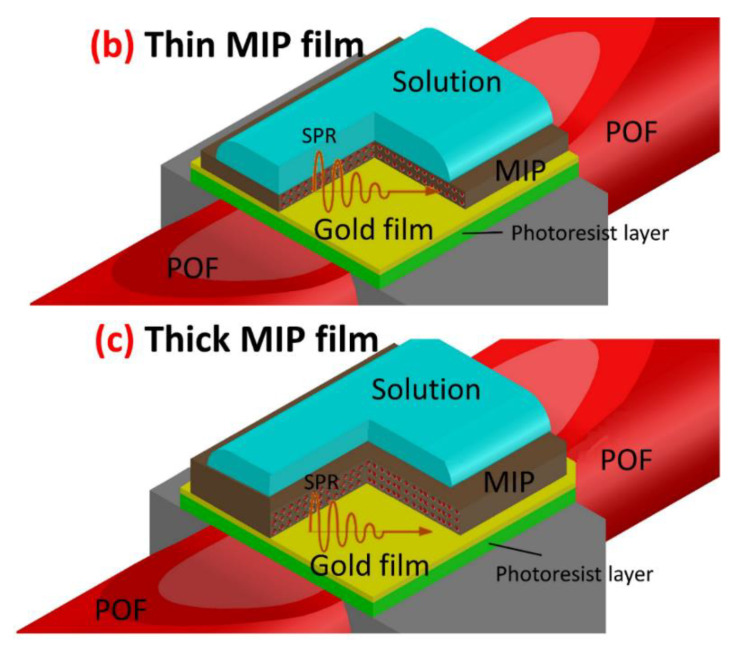
Optical-chemical sensor system: (**a**) outline of the experimental setup; (**b**) surface plasmon resonance- plastic optical fiber (SPR-POF) platform covered by a thin molecularly imprinted polymer (MIP) layer; (**c**) SPR-POF platform covered by a thick MIP layer. The grey block represents the resin support (1 cm × 1 cm × 1 cm) in which the POF is embedded.

The measurement apparatus consisted of a halogen lamp (HL–2000–LL, Ocean Optics) and a spectrometer connected to a PC (USB2000+UV–VIS spectrometer, Ocean Optics). The white light source presented an emission range from 360 nm to 1700 nm, whereas the spectrometer had a detection range from 350 nm to 1023 nm. The transmission spectra and data values were displayed online on the computer screen and saved by Spectra Suite software (Ocean Optics, Dunedin, FL, USA) [36]. An outline of the experimental setup based on spectral interrogation is reported in Figure 1a.

### 2.2. Preparation of the Fiber Optic Platform

The optical platform was based on a multimode POF with a characteristic D-shaped sensing region, obtained by erasing the cladding and partially the core of the POF, held in a specially designed resin support, which produced a flat surface (see Figure 1) [13,14,36]. One half of the fiber was erased and the exposed POF core was 1 cm long. A multilayer structure was built up over the exposed core with a buffer layer (a photoresist of high refractive index with respect to the core, 1.5 μm thick), a thin metal film (gold, 60 nm thick) and, finally, an MIP layer as a specific chemical receptor for 2-FAL detection. Figure 1b,c show two typical sensing regions obtained by two MIP layers with different thickness.

In particular, the buffer layer (Microposit photoresist, MicroChemCorp., Westborough, MA, USA) was deposited on the exposed core, taking advantage of the flat shape by dropping and spinning at 6000 rpm. The so obtained layer was 1.5 μm thick and the gold layer was deposited over it by sputtering (SCD 500, Leica Microsystems, Wetzlar, Germany), forming a nanofilm 60 nm thick.

### 2.3. Preparation of the Specific MIP Layer

The prepolymeric mixture was composed of the reagents at molar ratio 1 (2-FAL):4 (MAA):40 (DVB) according to the method previously described [14]. The cross-linker divinylbenzene (DVB) was also used as the solvent in which the functional monomer (methacrylic acid, MAA) and the template, 2-FAL, were dissolved. The mixture was uniformly dispersed by sonication and de-aerated with nitrogen for 10 min. Then, the radical initiator AIBN (23 mg/mL of prepolymeric mixture) was added to the mixture.

The MIP layer was prepared directly over the flat part of the platform, dropping a small volume (about 50 μL) of the prepolymeric mixture on the platform maintained in a flat position with the help of the resin support. The prepolymeric mixture expanded spontaneously to cover the erased surface of the POF and the surface of the holder. The whole structure was spun at a given spin rate, typically at 1000 rpm for 2 min, and then placed in an oven for 16 h at 80 °C for the thermal polymerization in air [13,14,19]. Finally, the template and oligomeric polymer fragments were removed by repeated washings with 96% ethanol.

Figure 1b,c show a schematic view of SPR-POF probes, covered by MIP layers of different thicknesses, in which the penetration of the plasmonic wave in the liquid above, in the case of thin and thick MIP layers, is schematically displayed. The layers with different thickness could be obtained by spinning the prepolymeric mixture at different rates, or by depositing multiple layers of MIP.

### 2.4. Measurement in a Drop

The flat surface of the described optical platform makes it possible to perform the measurement in a drop simply deposited over the flat surface. The platform was fixed in a mini holder, which was purposely designed to keep the sensing surface, embedded in the resin block, in a flat position. A sample drop (50 μL) was deposited over the flat part of the sensor, allowed to expand over the sensing surface and the support, and equilibrated for 5 min. During this time, the drop over the surface maintained its shape due to the MIP surface’s hydrophobicity, mainly constituted by DVB.

The concentration of 2-FAL in the sample only influenced the refractive index (RI) of the polymer in contact with the gold layer since the concentration of 2-FAL in the sample was too low to affect the solution’s RI. Any RI variation related to the matrix, the so-called “bulk effect”, had to be avoided or corrected.

As schematically shown in Figure 1b,c, this aspect is particularly relevant when very thin MIP layers are considered, i.e., thinner than the plasmonic wave penetration in the dielectric. In that case, the Δλ must always be measured in solution with the same RI. If the sample and the standard solutions have the same RI, the spectra can be recorded directly in the drop of the liquid sample positioned over the sensing layer, but when RI is different, the solvent exchange method proposed for SPR measurements in serum (*n* = 1.348 RIU at 600 nm [37]) in flowing conditions [38] must be applied.

In the static condition required by the determination in a drop here considered, a small volume of sample (40 µL) was dropped over the flat part of the sensor and equilibrated for 10 min as in the direct measurement method, but without recording the spectrum and measuring Δλ. The sample solution was eliminated by suction, and the platform was washed with 40 µL of water. After the washing step, 40 µL of water was deposited over the MIP to record all the spectra with the same bulk liquid overlying the receptor layer. In this way, only the RI change induced by the binding of the analyte was detected, without any possible perturbation induced by the bulk refractive index of the examined sample. The transmission spectra in water were normalized to the spectrum obtained with the corresponding sensor (SPR-MIP) in air (reference spectrum).

As a matter of fact, in the reference spectrum acquired on SPR-MIP with a thin MIP layer, no plasmon resonance was excited in the operative refractive index range of the platform here described [36].

### 2.5. Standardization Curves

The standardization curves were obtained by plotting the variation of the resonance wavelength in the normalized transmission spectra (Δ*λ*) vs. the concentration of 2-FAL. Δ*λ* was calculated respect to the resonance wavelength of a blank solution, i.e., a solution with the same composition of the sample but not containing the analyte of interest.

With the limited number of receptor sites in this kind of sensor, the response was linear only in very small concentration ranges. Therefore, the standardization curves were modeled by an equation deriving from the Langmuir adsorption isotherm [14,19,20], assuming that the signal is directly proportional to the amount of the template in the sensing layer (Δ*λ* = *k g c*_Aint_):

(1) $$\Delta \lambda =\frac{k\;g\;c_{\mathrm{int}}K_{\mathrm{aff}}\left[A\right]}{1+K_{\mathrm{aff}}\left[A\right]}=\frac{\Delta \lambda_{\max}K_{\mathrm{aff}}\left[A\right]}{1+K_{\mathrm{aff}}\left[A\right]}$$

[A] is the concentration of the analyte in the sample solution. *K*_aff_ (in mg^−1^ L) is the affinity constant of the adsorption equilibrium, *c*_int_ is the concentration of the specific sites of the MIP (in mg g^−1^), g is the polymer mass (in grams). Δ*λ*_max_ = *k*·*g·c*_int_ is the maximum Δ*λ* at high concentration of the analyte (i.e., when the analyte saturates all the specific sites).

Equation (1) can be applied as a standardization curve if the analyte concentration at the equilibrium in the sample, [A], is equal to the total one (*c*_A_), i.e., when the concentration of the analyte adsorbed is negligible.

The parameters of Equation (1) were obtained by Solver, the Microsoft Excel add-in program.

Once the parameters are known, the concentration of the analyte can be evaluated in the whole detection range from the measured Δ*λ*:

(2) $$\left[A\right]=c_{A}=\frac{\Delta \lambda }{K_{\mathrm{aff}}\cdot \left(k\cdot g\cdot c_{\mathrm{int}}-\Delta \lambda \right)}$$

## 3. Results

### 3.1. Response of the SPR-Bare Platforms to the Refraction Index Variation

**Figure 2:**
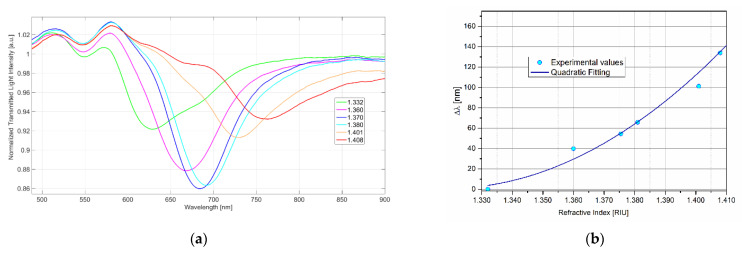
(**a**) Transmission spectra of the MIP-bare platform in water–glycerol solutions with different refractive index (RI). (**b**) Variation of the resonance wavelength with the RI of the overlying liquid.

The response of the SPR platforms, not derivatized with MIP (SPR-bare), here considered, has been checked as previously proposed [36]. In particular, the resonance wavelengths of the platform in liquid media of different RI, measured by an Abbe refractometer, were registered. As an example, the spectra of water–glycerol solutions with different RI are reported in Figure 2a. The response curve Δ*λ* (referring to pure water) against RI is shown in Figure 2b.

The sensitivity (δΔ*λ*/δRI) is significantly different from zero in the whole RI range, and it increases at increasing RI (Figure 2b). Nevertheless, for short RI ranges, the curve can be assumed to be linear. At increasing RI, the peak is shifted to higher wavelength values and becomes larger and flatter, making the acquisition of the minimum less accurate and precise. For this reason, the best RI of the dielectric layer in contact with the resonant surface must be a compromise between the highest sensitivity and precision.

### 3.2. MIP Layer Analysis

**Figure 3:**
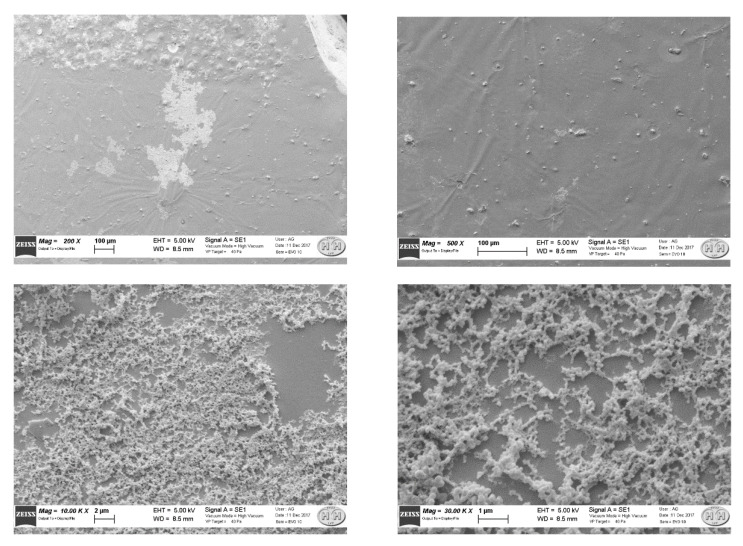
SEM images of different points at the surface of the sensing part.

The SEM image reported in Figure 3 shows the gold surface modified with MIP polymerized in situ, where the presence of polymer aggregates of small particles can be seen. This SEM image is relative to a sensing platform in which the MIP layer has been obtained by spinning the prepolymeric mixture at high velocity (about 1000 rpm). It shows that at the micro level, the MIP film does not entirely cover the gold surface, so in some way it reproduces the surface derivatized with a bioreceptor, i.e. a large biomolecule.

**Figure 4:**
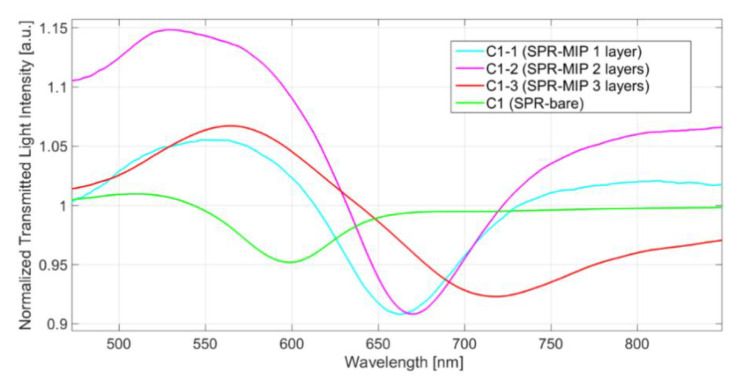
Transmission spectra of C1 (SPR-MIP) platform, with one, two, or three MIP layers, in water, normalized on the spectrum of the corresponding platforms in air.

Figure 4 reports the comparison between the SPR spectrum in water of a bare platform (SPR-bare) with that of the same platform modified with multiple layers of MIP (SPR-MIP). The MIP made the RI of the region overlying gold higher than pure water, as proven by the resonance wavelength shift towards higher values (red shift). The SPR spectra in Figure 4 are relative to a set of sensors prepared by depositing successively one, two, or three layers of MIP on equal platforms, as reported in Table 1. Water (*n* = 1.332 RIU) was the liquid over the platform. The example in Figure 4 shows that the resonance wavelength shift (Δ*λ*) with respect to that of pure water depends on the amount of MIP deposited. The resonance wavelengths are reported in Table 1.

By increasing the number of MIP layers, the resonance wavelength increases, and a shift to higher wavelengths (red shift) is observed (from 663.0 nm to 718.3 nm). Even with three layers deposited (C1-3), the resonance wavelength (718.3) is lower than the maximum wavelength useful for measurement.

**Figure 5:**
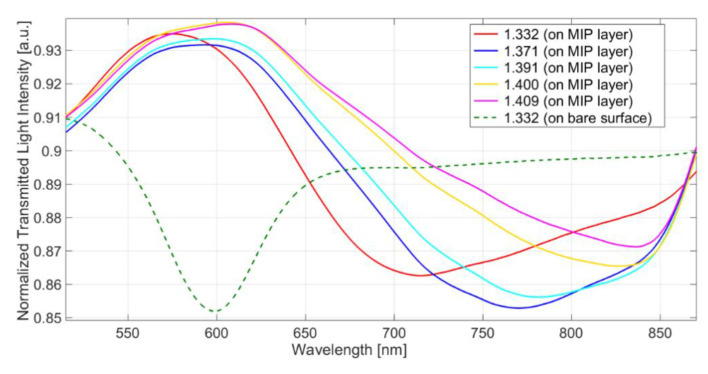
Comparison of the spectra of an SPR-MIP sensor (C1-3) in water–glycerol solutions with different refractive indices and the spectrum of an SPR-bare platform in water.

In order to verify if the sensor with three layers of MIP is still sensitive to the RI of the overlying liquid, the SPR transmission spectra in water–glycerol solutions with different refractive indices, positioned like a drop over the MIP (bulk solution), are reported in Figure 5.

The resonance in water of the SPR-MIP platform is at 715 nm (see Figure 4), with a shift of 115 nm compared to the resonance of SPR-bare in water, indicating that the amount of MIP layer is relatively large. However, a shift to higher wavelengths is observed when liquids with RI higher than water substitute water. This behavior demonstrates that the MIP layer is not so thick as to completely include the plasmonic wave, as schematically illustrated in Figure 1b,c.

### 3.3. Sensor Response in Water and in Wine-Mimicking Solution

**Figure 6:**
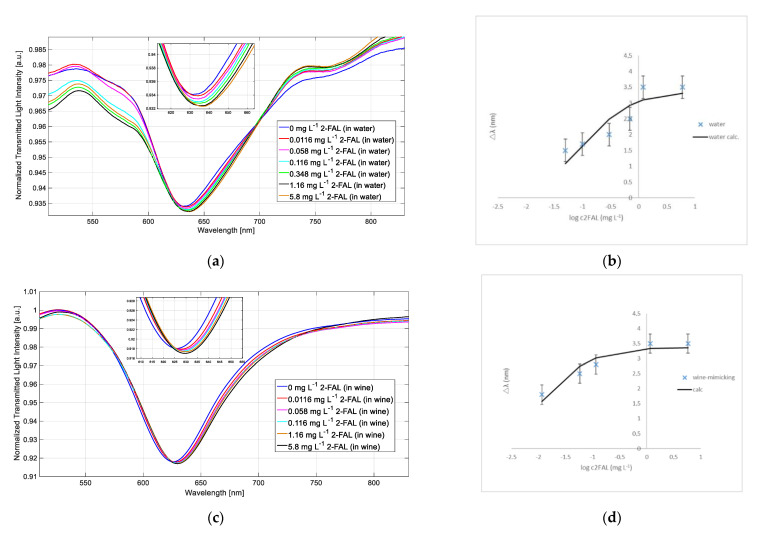
(**a**) SPR spectra of the SPR-MIP sensor at different 2-furaldheide (2-FAL) concentrations in water; (**b**) SPR wavelength shift vs. concentration of 2-FAL in a water solution and the calculated continuous curve; (**c**) SPR spectra of the SPR-MIP sensor at different 2-FAL concentrations in wine-mimicking solution; (**d**) SPR wavelength shift vs. concentration of 2-FAL in wine-mimicking solution and the calculated continuous curve. Vertical bars in (**b**,**d**) correspond to the standard deviations (error bars).

Water is a well-suited solvent for measurements with the platforms here described since its RI matches the operative range for the proposed SPR sensors [36]. The spectra can be recorded directly in the sample, since 2-FAL standards in water are easily prepared. When the 2-FAL concentration increases, the SPR wavelength is shifted to higher values, as seen in Figure 6a. As an example, a standardization curve of 2-FAL in water is shown in Figure 6b, and is obtained by reporting the wavelength variation Δ*λ* vs. 2-FAL concentration. The experimental data are well fitted by Equation (1), as expected when the sorption takes place according to the Langmuir model. A plateau corresponding to Δλ_max_ is obtained for concentrations higher than 1 mg·L^−1^ due to the receptor’s sites saturation. The parameters, evaluated by a non-linear regression method, are *K*_aff_, 9.4 L·mg^−1^ (9.0 10^5^ M^−1^) and Δ*λ*_max_ = 3.3 (0.447) nm. The sensitivity at low concentration (i.e., in the linear part of the curve) is 31.6 nm·mg^−1^·L. The saturation was reached at about 1 mg·L^−1^.

The response of the sensor to 2-FAL concentration in wine-mimicking solution is reported in Figure 6c,d. In this case, the spectra were recorded in water, after equilibration with the sample and washing, according to the method of the solvent exchange.

The dose–response curve (Δ*λ* vs. 2-FAL concentration) in wine-mimicking solution followed the Langmuir model (see Equation (1)) and the parameters obtained are: *K*_aff_ = 75.6 L·mg^−1^ and Δ*λ*_max_ = 3.3 (0.247) nm. The sensitivity at low concentration is 254.9 nm·mg^−1^·L, and the limit of detection (LOD) is 0.004 mg·L^−1^**.** The saturation was reached at about 0.6 mg·L^−1^.

The affinity constant is about 8 times higher in wine-mimicking solution than in pure water; consequently, the LOD is lower than in water.

### 3.4. Determination of 2-FAL in a White Wine Sample

**Figure 7:**
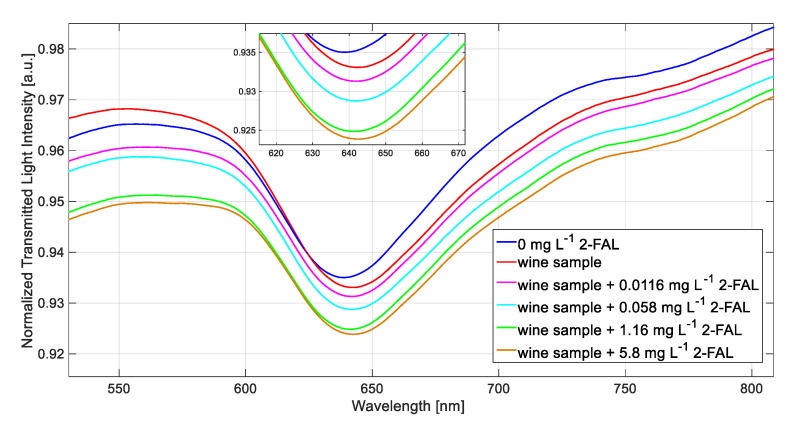
SPR spectra obtained by the SPR-MIP sensor in wine sample.

The sample considered was a white wine in a tetra pack container (WW) purchased in a local supermarket, in which a concentration of 2-FAL of 0.1 ppm was found by an HPLC method. The SPR spectra were obtained as reported in the experimental part, using water as the liquid in which the spectra were finally recorded (bulk liquid). This method had to be applied since the exact RI of the white wine is unknown. Figure 7 shows the transmission spectra recorded in water of the considered WW sample, and with some standard additions of 2-FAL.

The variation of *λ*_ris_ from the blank (water) to the WW (2.8 nm) is due to the adsorption of 2-FAL on the MIP during the incubation with the sample. There is only a very slight variation of *λ*_ris_ in response to the standard additions of 2-FAL to the sample. This behavior indicates that the imprinted sites are almost saturated by the 2-FAL originally present in the WW considered. In fact, the concentration calculated from Equation (1), using the parameters evaluated in wine-mimicking solution, is 0.107 mg L^−1^, near to the approximate saturation concentration, in acceptable agreement with the value found by the HPLC method (0.1 mg L^−1^).

## 4. Discussion

In the SPR-POF sensing apparatus here considered, the operative RI ranges were from about 1.33 to 1.41 (see Figure 2). In aqueous matrices with low RI, an RI in this range is obtained when a thin MIP layer is deposited on the gold surface so that the plasmonic wave partially penetrates in the overlying aqueous medium, as shown in Figure 1b. The preparation of the MIP layer reported in the experimental part allowed this goal to be achieved, i.e., having an RI of the layer (MIP plus water solution) in the suitable range. The resonance wavelength shift of the sensor with MIP in water, with respect to that of the bare platform in water, was around 50–150 nm. Similar shifts were previously observed in sensors with MIP’s receptor. For example, a value as high as 150 nm in sensors for TNT [13] and nicotine [19], and about 80 nm in a 2-FAL sensor [27] were obtained. Lower Δ*λ* was observed in the case of a monolayer of molecular receptor as in the case of an aptamer [17] or a metal ligand [16,19], confirming that the shift is higher when a higher amount of receptor is present at the resonant surface. As shown in the present investigation (Figure 2), the resonance wavelength should not exceed about 800 nm, so that an MIP layer that is not too thick must be used.

On the other hand, this makes the sensor sensitive to the RI of the overlying liquid, which thus must be carefully controlled.

For this reason, when measuring a real life sample, for example wine, using the sensing method proposed, any RI variation due to the matrix composition must be avoided. This aspect is relevant when thin MIP layers are considered, i.e., thinner than the plasmonic wave penetration in the dielectric. In that case, the Δ*λ* must always be measured in solution with the same RI. The matrix effects are avoided by equilibrating the sample with the sensor and then changing the sample with a solvent with a proper RI (for example, an aqueous buffer), for recording the spectrum and measuring Δ*λ*. In the present work, to demonstrate the method’s effectiveness, pure water was selected as the liquid for registering the spectrum for the analysis of 2-FAL in a white wine sample. An alternative approach could be applied, using a much thicker MIP layer; however, in this case, the RI of the dielectric over gold could not be suitable for the measurements since the resonance wavelength could be outside the operative range.

## 5. Conclusions

The proposed platform, based on D-shaped POF and an MIP as the receptor layer, has been demonstrated to be particularly suited for measurements in aqueous matrices since the RI of the layer in contact with the resonant surface (gold) matches the measurable RI range. On the other hand, this strongly depends on the MIP layer’s thickness, at least for the MIP here considered.

It has been found that the “wine” matrix has a large effect on the SPR signal since the response of the sensor to 2-FAL is different in water and wine-mimicking solution. The affinity constant is one order of magnitude higher in wine than in water, and consequently, a lower LOD is obtained, reaching interesting values for the determination of 2-FAL in wine.

The method developed for the determination of 2-FAL in wine could have a high practical interest; on the one hand, for the possible toxic and carcinogenic effects of furanic aldehydes, particularly 2-FAL, on human beings and, on the other hand, for their impact on the aroma of food. Interestingly, the sensor here developed could be easily adapted to the analysis of other furanic compounds.

## Figures and Tables

**Table 1:** Resonance wavelength of sensors with multiple MIP layers. Formation of each MIP layer: 40 µL of prepolymeric mixture spun at 300 rpm. Normalization on the corresponding platform in air.

Type of Sensor	N. of MIP Layers	*λ*_ris_ in Water [nm]	Δ*λ* (SPR-MIP in Water Minus SPR-Bare in Water) [nm]
C1 (SPR-bare)	0	597.7	0
C1-1 (SPR-MIP)	1	663.2	65.5
C1-2 (SPR-MIP)	2	672.4	74.7
C1-3 (SPR-MIP)	3	718.3	120.6
